# Genital Lesions Masquerading as Condyloma Acuminata: A Case Series Report

**Published:** 2019-06

**Authors:** Parvin MANSOURI, Zahra S. NARAGHI, Maliheh AMANI, Reza CHALANGARI, Katalin MARTITS-CHALANGARI, Nikoo MOZAFARI

**Affiliations:** 1. Skin and Stem Cell Research Center, Tehran University of Medical Sciences, Tehran, Iran; 2. Department of Dermatopathology, Tehran University of Medical Sciences, Tehran, Iran; 3. Skin Research Center, Shahid Beheshti University of Medical Sciences, Tehran, Iran; 4. Kassir Dermatology, Dallas, Texas, USA

**Keywords:** Syringoma, Lymphangioma, Vestibular papillomatosis, Genital warts

## Abstract

Herein we report three married women referred to Dermatology Clinic of Loghman Hakim Hospital, Tehran, Iran in 2017 for evaluation and treatment of genital warts. Two patients were complaining of flat-topped papules on their labia major and the third one was presented with asymptomatic papillary projections on her vestibule and inner aspect of both labia minora. Histological examination revealed the diagnosis of syringoma, lymphangioma circumscriptum (LC) and vestibular papillomatosis respectively. Familiarity with these uncommon conditions which clinically mimic genital warts helps to prevent labeling a patient with sexually transmitted disease before histological confirmation and prevent unnecessary treatment.

## Introduction

Giving a diagnosis of genital warts could be extremely stressful for faithful partners as they may feel they have been betrayed. It is very important to precisely confirm the diagnosis, before labeling a patient with a sexually transmitted disease. Here we report three patients referred to Dermatology Clinic of Loghman Hakim Hospital, Tehran, Iran in 2017 for evaluation and treatment of genital warts; while they were proven to have syringoma, lymphangioma circumscriptum (LC) and vulvar papillomatosis by histologic examination.

### Case 1

**Fig. 1:**
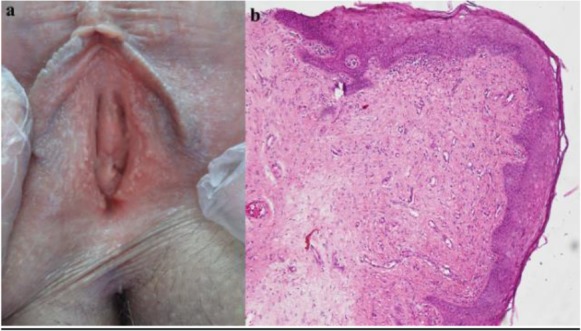
**(a);** Multiple fine, pink papillary projections, located into the inner aspect of the labia minora. **(b):**There are thickening and hyperplasia of the nonkeratinizing squamous epithelium rich in glycogen, overlying central fibrovascular core (H&E 10)

A 45 yr old woman was referred by a gynecologist for evaluation of suspected genital warts. The patient was not aware of these lesions until they were noticed by her gynecologist on a routine examination. Our patient was a healthy married multiparous woman with no history of extramarital sexual contacts. She gave no history of genital lesions on her husband. Examination identified soft digitate mucous colored papules on the vestibule and inner aspect of both labia minora (Fig. 1a).

A biopsy was performed showing a normal epidermis overlying a papillomatous lesion with fibrovascular stroma. No koilocytes were seen. Immunohistochemical staining for human papillomavirus (HPV) was negative. The findings were consistent with a diagnosis of vulvar vestibular papillomatosis (Fig. 1b).

### Case 2

**Fig. 2:**
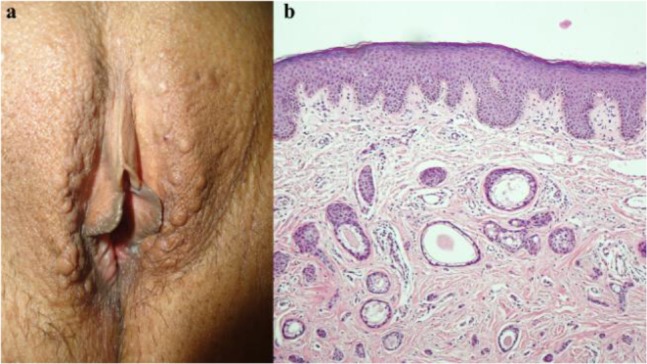
**(a):** multiple skin-colored vulval papules. **(b):** Circumscribed tumor composed of ducts and strands embedded in a fibrous stroma. Some of the ducts are in the form of epithelial strands with a tadpole or comma-like appearance (H&E 10)

A 39 yr old woman was referred to the Dermatology Clinic with 2 yr history of increasing asymptomatic multiple skin-colored lesions in her vulva. She had been diagnosed as genital warts. Physical examination revealed multiple discrete, soft, flat-topped papules measuring 1 to 3 mm in diameter on the outer labia majora (Fig. 2a). The labia minora were normal and there were no similar lesions at any other sites. A punch biopsy revealed numerous ductal structures dispersed within a fibrous stroma. The ducts lined by two rows of epithelial cells; some showed “comma-like tail” or “tadpole” appearance (Fig. 2b). Subsequently, the patient was diagnosed with syringoma.

### Case 3

**Fig. 3:**
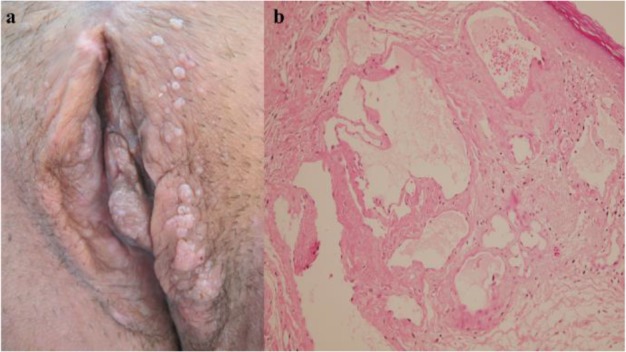
**(a):** Multiple clusters of grayish white papules on labia major and clitoris. **(b):** in the superficial and deep dermis numerous dilated spaces contained eosinophilic homogenous material and few erythrocytes are seen (H& E 20)

A married 44-yr-old woman presented with progressive verrucous vulvar lesions for three years. She also was complaining of pruritus and burning sensation. Her past medical history was unremarkable and there were no similar lesions on her husband. On physical examination, there were clusters of grayish white papules varying from 3 to 7 mm in size on labia major and clitoris (Fig. 3a). She had been treated as a genital wart with podophyllin and cryotherapy without any benefit. Subsequently, a punch biopsy was done. Histological examination revealed large, irregular cystic dilatations of vascular spaces, contained proteinous fluid and red cells (Fig. 3b). Positive immunohistochemistry (IHC) staining for (D2-40) confirmed the diagnosis of Lymphangioma circumscriptum (LC).

Informed consent was taken from all the patients for publication of their images and Ethical Committee of the Shahid Beheshti University of Medical Sciences approved the study protocol.

## Discussion

### Vestibular papillomatosis

vulvar vestibular papillomatosis (VP) is regarded as a normal variant in the vulvar vestibule architecture; with a prevalence between 5.1% to 33% (1). The lesions most often are asymptomatic though pruritus, pain, burning, and dyspareunia has been noted among some patients (2). Clinically VP appears as multiple smooth mucosal papillary projections. They symmetrically cover the inner labia minora and vaginal introitus (2). VP clinically resemble wart, while there are many studies that showed no relationship between VP and HPV infection (3, 4). Some clinical characteristics were proposed to facilitate distinguishing VP from condyloma acuminatum (3). VP typically are soft, regular and mucous colored. They distributed in a symmetrical and linear array, limited to the inner labial minora and vaginal introitus. The bases of individual projections remain separate. Whereas condyloma accuminata are firm and in varying colors; pink, white, or red. They are randomly distributed across the vulvar mucosa. Filiform projections of condyloma accuminata tend to coalesce into shared bases and in spite of VP, in most cases of condyloma accuminata sharp whitening can be observed using acetic acid test. Attention to these characteristics during the physical examination can help correct diagnosis and prevent unnecessary treatment.

### Syringoma

Syringomas are benign adnexal tumors of eccrine origin. They usually present as multiple, soft, skin-colored papules. They often involve the periorbital area or can be present in an eruptive form involving neck, chest and upper arms (5). However, localized vulvar syringoma is considered to be rare (6). They are generally asymptomatic but may be cosmetically disturbing for patients. No treatment is necessary other than for cosmetic concerns. Treatment options include surgical excision, electrodesiccation, cryo-surgery and carbon dioxide laser (7). All treatments are associated with risk of recurrence, scaring and pigmentary changes (7).

### Lymphangioma Circumscriptum

Lymphangioma circumscriptum (LC) is a subtype of vascular malformation, characterized by aggregation of relatively small and abnormal lymphatic channels protruding through the epidermis (8). LC can be congenital or acquired. The acquired form usually occurs after impaired lymphatic flow following surgery, radiotherapy or malignant or infectious process. Sites of predilection in primary form, include the proximal parts of limbs, trunk, and oral cavity. Vulvar involvement is uncommon and is often acquired (8). While in our case history of surgery and investigation for Tuberculosis or malignancy were negative. Our case seems to be a late manifestation of the congenital variant. The most preferred treatment option for LC is surgical excision however recurrence is common (9). Ablative modalities such as carbon dioxide laser, radiofrequency ablation and cryo-therapy are accompanied with short term cure and high recurrence rate (8, 9). To prevent a recurrence, the deep feeding lymphatic cisterns should be removed. Although radiotherapy has been successful in one patient with vulvar LC (10), it may cause premature ovarian failure and secondary malignancy.

## Conclusion

There are some entities which may be clinically indistinguishable from genital warts. A biopsy is essential to confirm the diagnosis. The focus in this case report includes the familiarity with these uncommon conditions, preventing labeling a patient with the sexually transmitted disease and preventing unnecessary treatment.

## Ethical considerations

Ethical issues (Including plagiarism, informed consent, misconduct, data fabrication and/or falsification, double publication and/or submission, redundancy, etc.) have been completely observed by the authors.
